# Cell‐Free Protein Synthesis for the Screening of Novel Azoreductases and Their Preferred Electron Donor

**DOI:** 10.1002/cbic.202200121

**Published:** 2022-06-16

**Authors:** Jascha Rolf, Anna Christina Reyes Ngo, Stephan Lütz, Dirk Tischler, Katrin Rosenthal

**Affiliations:** ^1^ Department of Biochemical and Chemical Engineering Chair for Bioprocess Engineering TU Dortmund University Emil-Figge-Str. 66 44227 Dortmund Germany; ^2^ Microbial Biotechnology Faculty of Biology and Biotechnology Ruhr-Universität Bochum Universitätsstr. 150 44780 Bochum Germany

**Keywords:** azo bonds, azo dyes, biocatalysis, CFPS, enzyme screening, NADH, NADPH

## Abstract

Azoreductases are potent biocatalysts for the cleavage of azo bonds. Various gene sequences coding for potential azoreductases are available in databases, but many of their gene products are still uncharacterized. To avoid the laborious heterologous expression in a host organism, we developed a screening approach involving cell‐free protein synthesis (CFPS) combined with a colorimetric activity assay, which allows the parallel screening of putative azoreductases in a short time. First, we evaluated different CFPS systems and optimized the synthesis conditions of a model azoreductase. With the findings obtained, 10 azoreductases, half of them undescribed so far, were screened for their ability to degrade the azo dye methyl red. All novel enzymes catalyzed the degradation of methyl red and can therefore be referred to as azoreductases. In addition, all enzymes degraded the more complex and bulkier azo dye Brilliant Black and four of them also showed the ability to reduce *p*‐benzoquinone. NADH was the preferred electron donor for the most enzymes, although the synthetic nicotinamide co‐substrate analogue 1‐benzyl‐1,4‐dihydronicotinamide (BNAH) was also accepted by all active azoreductases. This screening approach allows accelerated identification of potential biocatalysts for various applications.

## Graphical abstract

**A screening approach** for azoreductases was developed, including cell‐free protein synthesis of an enzyme library and a multi‐well microplate assay. Applicability to known and novel enzymes was demonstrated, and preferred co‐substrates for each enzyme were evaluated. Five new and yet uncharacterized azoreductases were identified. Furthermore, it was shown that all azoreductases accept the co‐substrate BNAH as an electron donor.

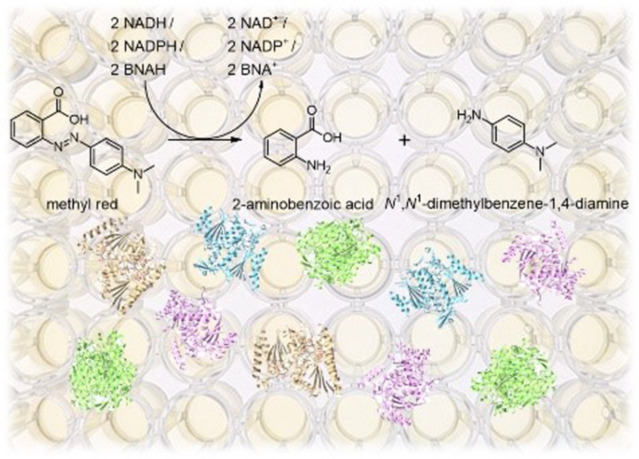

## Introduction

**Figure 1:**
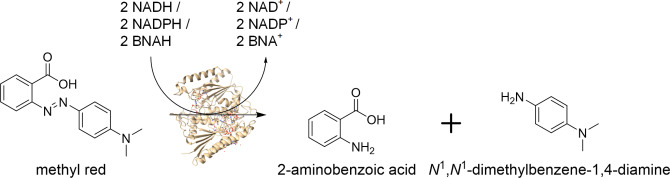
Schematic illustration of the catalyzed cleavage of the azo bond by an azoreductase using different co‐substrates as electron donors (enzyme crystal structure of AzoRo, PDB ID: 7AWV).

Azo dyes are characterized by containing one or more azo bonds (R−N=N−R′). They are widely used to treat textiles, leather, and paper and find application in the cosmetics and pharmaceutical industries.^[1]^ Due to their widespread use, azo dyes often contaminate wastewater and pose a threat to the environment. Even low concentrations cause aesthetic pollution and prevent the penetration of light through water. In addition to their visual impact, azo dyes also have negative effects in terms of total organic carbon and chemical oxygen demand, which are indicative of organic pollutants in waste water.^[2]^ Many synthetic azo dyes and their metabolites are toxic, carcinogenic, and mutagenic, resulting in potential health hazards to humans.^[3]^ Key enzymes in the biodegradation of azo dyes are the azoreductases, which catalyze the reductive cleavage of azo bonds. The enzymes are widely found in bacteria and act on numerous azo dyes, which allow various unique applications.^[4]^ The reduction of one azo bond by azoreductases requires two molecules of nicotinamide co‐substrate (Figure 1).

Many azoreductases belong to the family of flavin mono nucleotide (FMN)‐dependent NADH : quinone oxidoreductases, but there are also other enzymes such as cytochrome P450s in mammalians that catalyze the reduction of azo bonds.^[5]^ Some azoreductases do not prefer any type of electron donor and accept both naturally occurring ones, nicotinamide adenine dinucleotide (NADH) and nicotinamide adenine dinucleotide phosphate (NADPH),^[6]^ while others show strict preferences.^[7]^ To avoid the stoichiometric addition of these expensive coenzymes, several NAD(P)H regeneration systems can be used, for example in combination with dehydrogenases.[^8^, ^9^] Alternatively, a less expensive reductant, the synthetic nicotinamide co‐substrate analogue 1‐benzyl‐1,4‐dihydronicotinamide (BNAH), was also shown to be accepted by an azoreductase.^[10]^ These kind of biomimetics can be an attractive alternative to natural co‐substrates not only in terms of cost but also in terms of stability and reactivity.^[11]^ The flavin‐containing oxygen‐insensitive azoreductase from *Rhodococcus opacus* 1CP was identified as NADH, NADPH, and BNAH‐accepting and catalyzing the degradation of methyl red, one of the azo dyes most commonly accepted by azoreductases.[^10^, ^12^] Azoreductases can be classified phylogenetically into four main clades based on their primary sequences.^[7]^ On this basis, however, no statement can be made as to which co‐substrate is accepted by the enzyme. For the identification of further azoreductases and verification of their co‐substrate acceptance, novel enzymes need to be screened, isolated, and tested *in vitro*. However, conventional heterologous expression in a host organism is costly and laborious if a large number of genes are to be studied. In contrast, cell‐free protein synthesis (CFPS) provides a tool for the rapid synthesis of enzymes. It is a widely used method for the fast transcription and translation of genes with a broad range of applications.[^13^, ^14^, ^15^] CFPS can avoid time‐consuming steps, such as expression strain construction, culture growth, and protein purification. Thus, protein concentrations at a milligram per milliliter scale can be achieved within a few hours of synthesis time. Due to its open nature, CFPS offers the possibility of easy manipulation of the synthesis conditions and is, therefore, a valuable platform for the synthesis of difficult‐to‐express proteins, such as proteins with a toxic effect on host cell metabolism^[16]^ or proteins that tend to be synthesized in insoluble form.^[17]^ Thus, the applicability of CFPS for protein screenings and characterizations has already been shown in several studies.[^18^, ^19^, ^20^]

In this study, we aimed to develop a screening setup using CFPS in combination with a subsequent *in vitro* activity assay in a multi‐well microplate, which allowed the parallel screening of several putative genes. Thus, suitable candidates for a biotechnological application could be found quickly and easily. Therefore, different proteins termed putative azoreductases, but never biochemically characterized, were synthesized using CFPS and evaluated for their ability to cleave azo bonds in *in vitro* assays. Moreover, the acceptance of the different co‐substrates NADH, NADPH, and BNAH were tested in this setup for 10 enzymes with homologous primary sequences (Figure S1). We have shown for the first time that azoreductases can be expressed in active form in an *Escherichia coli*‐based CFPS system. Thus, we identified five previously unknown putative azoreductases that functionally degrade azo dyes. In addition, the co‐substrate specificity of active homologs was evaluated. Interestingly, the co‐substrate mimic BNAH allowed successful cleavage of the azo bond for all employed azoreductases. In future, this systematic screening approach will make it easy to identify further azoreductases of biotechnological importance in a short time.

## Results and Discussion

### Development of a CFPS screening setup for azoreductases

In the first step, the screening setup was developed using the azoreductase from *Rhodococcus opacus* 1CP.^[21]^ For consistency in naming, it will be referred to as *Ropa*AzoR in the following. The enzyme assay was adapted to the microplate scale from Qi *et al.*.^[21]^ The optimal synthesis conditions were evaluated to obtain active enzymes in sufficient amounts. The synthesis temperature for CFPS systems based on *E. coli* is often 30 to 37 °C due to the temperature optimum of the parent organism. The synthesis times at this temperature range from 2 to 8 h. Based on these parameters and own experiences,^[17]^ the synthesis conditions of 37 °C and 4 h were selected for the first experiments with an in‐house *E. coli* extract‐based CFPS system. Unfortunately, the high background activity of the *E. coli* extract rendered this synthesis system useless for subsequent methyl red degradation assays. Within a few seconds, the entire azo dye was degraded by endogenous *E. coli* enzymes. Therefore, no activity of *Ropa*AzoR could be determined. The high background activity can be explained by the already described azoreductase from *E. coli*, which is probably present in the CFPS system and is known to degrade methyl red.^[22]^

**Figure 2:**
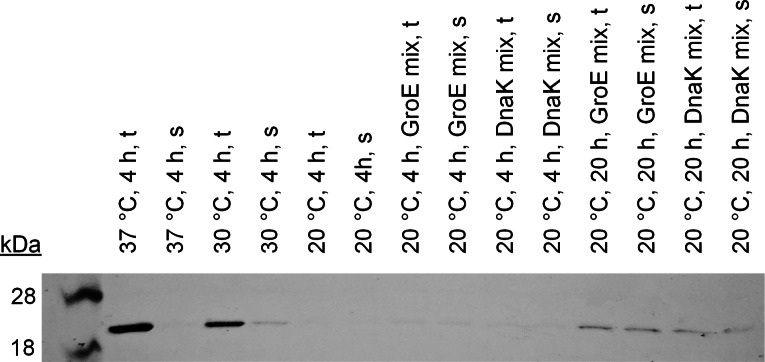
Western blot image of cell‐free synthesized *Ropa*AzoR with 10xHis tag (25.3 kDa) with PUREfrex2.0 at different synthesis parameters and with chaperone mixes. t: total protein fraction; s: soluble protein fraction.

For this reason, a purification of the proteins is necessary in any case for a cell‐based synthesis in *E. coli* as well as for the use of an *E. coli* extract‐based CFPS system. Since purification is not worthwhile due to the small volumes involved in CFPS, a CFPS system was chosen that was reconstructed exclusively from the essential elements of the translation system of *E. coli* and the T7 RNA polymerase for transcription. The so‐called protein synthesis using recombinant elements (PURE) system contains all building blocks required for mRNA and protein synthesis, such as nucleotides and amino acids.^[23]^ We successfully synthesized the enzyme at 37 °C, however, the enzyme showed no activity in the methyl red degradation assay. Analysis of the total and soluble protein fraction revealed that the azoreductase was detectable only in the total protein fraction and thus was not present in active form (verified by western blot, Figure 2). Some proteins synthesized with heterologous systems tend not to fold properly and therefore become insoluble.^[24]^ This issue can be addressed with different approaches.^[25]^ The most common strategies are exploring the choice of vector, host strain, expression conditions, use of fusion tags, and chemical or biological chaperones. These methods can also be transferred to CFPS. We decided to test, first, the reduction of the synthesis temperature and, second, the addition of chaperones to synthesize the highest possible fraction of soluble enzyme. To slow down the translation process, lowering the temperature is one of the simplest methods used in traditional cell‐based expression but also in CFPS. We tested different temperatures at 20, 30, and 37 °C for *Ropa*AzoR synthesis. The western blot showed only a very weak band for the soluble fraction in a synthesis at 30 °C (Figure 2). For 20 °C synthesis temperature, bands were barely visible in any fraction. This does not exclude the synthesis of active enzyme but may be caused by the small amounts that were formed.

In addition to the reduction of expression temperature, a common approach is the usage of molecular chaperones to improve the solubility of proteins. These methods can also be applied to CFPS systems to increase the expression yields of soluble proteins. For example, chaperone‐enriched cell extracts can be used[^17^, ^26^] or due to the open environment, the exogenous addition of molecular chaperones can be easily performed.[^27^, ^28^, ^29^] Two commercially available chaperone mixes, the DnaK and GroE mix, were tested for the synthesis of *Ropa*AzoR in this study. The DnaK mix consists of purified DnaK, DnaJ, and GrpE from *E. coli* with an optimized ratio. DnaK has ATPase activity and is stimulated by co‐chaperones, DnaJ and GrpE. DnaJ facilitates the ATPase activity of DnaK and can bind to a hydrophobic region of the synthesized protein. GrpE stimulates the ADP/ATP exchange rate of DnaK. The GroE mix consists of purified GroEL and GroES from *E. coli*. GroES affects the folding activity of GroEL by regulating its ATPase activity. Figure 2 elucidates that the synthesized enzymes are almost completely in the soluble fraction when chaperones were used at 20 °C. Especially, with an extended synthesis time of 20 h, detectable amounts of enzymes could be synthesized in the soluble fraction.

**Figure 3:**
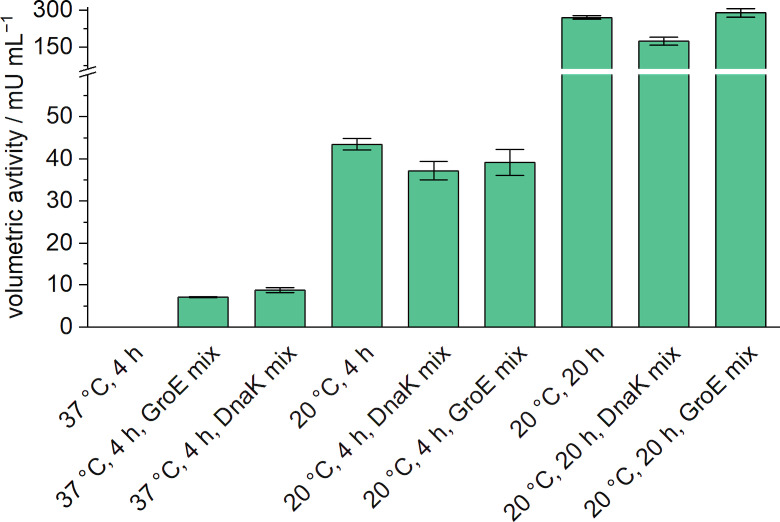
Volumetric activities of the synthesis mix with *Ropa*AzoR under different synthesis conditions. PUREfrex2.0 was used for CFPS for 4 to 20 h at 20 °C and 37 °C. Activity assays were carried out in a 96‐well microplate with a total volume of 100 μL, consisting of 10 % (v/v) CFPS‐reaction solution and the assay solution with following final concentrations: 100 mM sodium phosphate buffer (pH 6), 50 μM FMN, 30 μM methyl red, and 150 μM NADH. Reactions were incubated at 25 °C and the degradation of methyl red was monitored by UV‐vis spectrophotometry at 430 nm. Error bars are a result of duplicates. Duplicates are from two independent activity assays with enzymes derived from the same synthesis batch.

Subsequently, activity assays were performed with *Ropa*AzoR obtained from syntheses under various conditions, namely different temperatures, synthesis times, and the addition of chaperones (Figure 3). In these experiments, we also used chaperones for enzyme synthesis at 37 °C, and we tested the activity of enzymes synthesized at 20 °C without the addition of chaperones.

By using the respective chaperone mixes, active *Ropa*AzoR was synthesized, and substrate degradation was detected in the activity assay. Lowering the synthesis temperature also showed a positive effect. A synthesis temperature of 20 °C resulted in volumetric activities of 43±1 mU mL^−1^ of the CFPS mix. The volumetric activity of enzymes, which were synthesized at 20 °C without chaperones, was thus about 5 times higher compared to the syntheses with chaperones at 37 °C. Since it can be assumed that only small amounts of enzyme can be synthesized at 20 °C in the selected synthesis time of 4 h, longer synthesis times were also tested. An extension to 20 h resulted in an increase of the volumetric activity to 270±6 mU mL^−1^ (Figure 3). The addition of chaperones increased the activity in the 37 °C experiments but showed no beneficial effect in any experiments at 20 °C. Since the highest activity was achieved at a synthesis temperature of 20 °C and a synthesis time of 20 h, these parameters were used for the subsequent experiments and the screening of further azoreductases. The addition of the expensive chaperone mixtures could be omitted since they did not result in a beneficial effect under these synthesis conditions.

In a recent study, fusion proteins were constructed consisting of the *Ropa*AzoR and a formate dehydrogenase (FDH) from *Candida boidinii*.^[9]^ These constructs from the two biocatalysts showed lower activity than the azoreductase alone but were able to degrade azo dyes without the addition of NADH. Here, the activity was more affected when the azoreductase was placed at the N‐terminus of the fusion construct, indicating that the positioning and design of a bifunctional catalyst affect the activities. To test, if our CFPS‐based screening setup can also be used for such studies, we selected two fusion constructs consisting of *Ropa*AzoR and the FDH in different orientations. The fusion proteins have a molecular weight of about 70 kDa, more than 3 times the size of the azoreductase previously tested. The enzymes were synthesized under the same conditions as described before and tested in the subsequent activity assay for their methyl red degradation ability.

**Figure 4:**
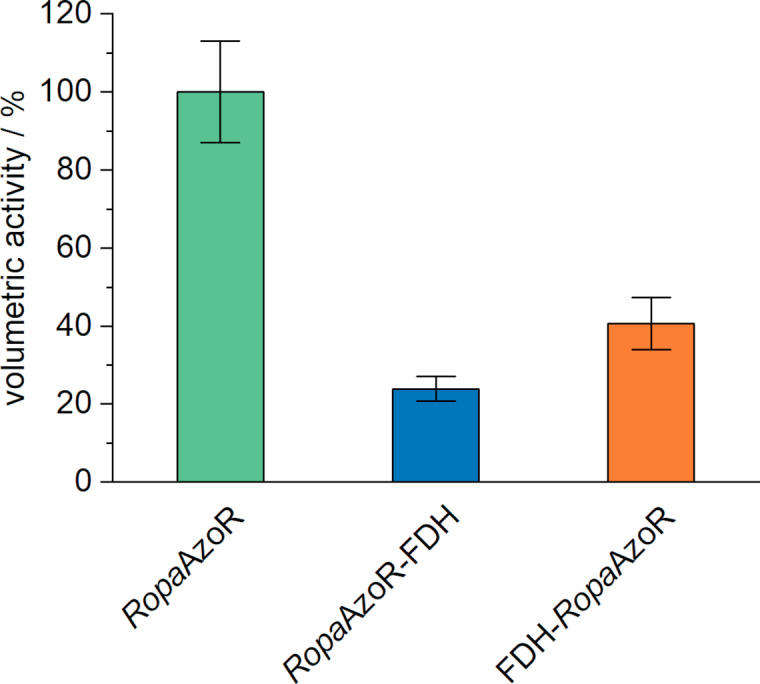
Volumetric activities for *Ropa*AzoR and fusion proteins consisting of *Ropa*AzoR and a formate dehydrogenase (FDH) from *Candida boidinii*. Values were normalized to *Ropa*AzoR, which corresponds to 100 % activity. Activity assays were carried out in a 96‐well microplate with a total volume of 100 μL, consisting of 10 % (v/v) CFPS‐reaction solution and the assay solution with following final concentrations: 100 mM sodium phosphate buffer (pH 6), 50 μM FMN, 30 μM methyl red, and 150 μM NADH. Reactions were incubated at 25 °C and the degradation of methyl red was monitored by UV‐vis spectrophotometry at 430 nm. Error bars are a result of duplicates. Duplicates are from two independent activity assays with enzymes derived from the same synthesis batch.

Both fusion proteins were synthesized in active form and were able to catalyze the cleavage of the azo bond in methyl red. The substrate depletion rate was lower than for the wild‐type azoreductase, resulting in volumetric activities of 24±3% and 41±7% for the C‐terminal and the N‐terminal tagged *Ropa*AzoR, respectively (Figure 4).

Our results indicate that the placement of the azoreductase at the N‐terminus leads to a greater loss of activity, confirming previous results where the position at the N‐terminus impaired the azoreductase activity.^[9]^ However, the remaining activity was about 5 %, which was even lower than in our study. Nevertheless, it was shown that these more complex constructs can also be synthesized in active form and that the results are comparable to *in vivo* synthesized and subsequently purified enzymes.

### Screening of putative azoreductases and their preferred electron donor

For the screening, in total 10 different proteins were selected, which are annotated on uniport.org as FMN‐dependent NADH : quinone oxidoreductases (Table 1).^[30]^ Five of them were already confirmed as azoreductases and can degrade methyl red under the consumption of NADH or NADPH. The other five proteins are inferred from homology and have never been expressed heterologously before. Four phylogenetic clades can be defined for azoreductases according to their primary sequence similarity.^[7]^ The selected proteins can be assigned to clades II and III except for *Ropa*AzoR, which cannot be assigned to one of the four clades (Figure S2). Actually, it seems to be an evolutionary intermediate from clade II to clade III. This needs to be studied in more detail for a clear assignment.

**Table 1:** Described and putative azoreductases employed in this study.

Protein	UniProt ID	Source organism	Clade	Reference
*Ropa*AzoR	A0A1B1KJ01	*Rhodococcus opacus* 1CP	II/III	AzoRo^[21]^
*Ecol*AzoR	P41407	*Escherichia coli*	III	AzoR^[22]^
*Bsub*AzoR	O32224	*Bacillus subtilis*	II	YvaB^[31]^
*Paer*AzoR	Q9I5F3	*Pseudomonas aeruginosa*	III	paAzoR1^[32]^
*Efae*AzoR	Q831B2	*Enterococcus faecalis*	II	AzoA^[33]^
*Styp*AzoR	P63462	*Salmonella typhimurium*	III	This study
*Rfer*AzoR	Q220J4	*Rhodoferax ferrireducens*	III	This study
*Plau*AzoR	Q7N511	*Photorhabdus laumondii* subsp*. laumondii*	III	This study
*Blat*AzoR	Q39M92	*Burkholderia lata*	III	This study
*Tvar*AzoR	Q3M1N6	*Trichormus variabilis*	III	This study

**Figure 5:**
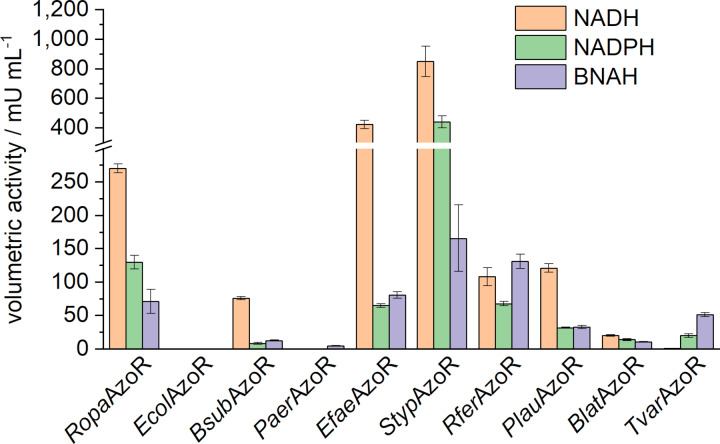
Volumetric activities for the selected azoreductases in combination with different co‐substrates. Activity assays were carried out in a 96‐well microplate with a total volume of 100 μL, consisting of 1 to 10 % (v/v) CFPS‐reaction solution and the assay solution with following final concentrations: 100 mM sodium phosphate buffer (pH 6), 50 μM FMN, 30 μM methyl red, and 150 μM NADH/NADPH/BNAH. Reactions were incubated at 25 °C and the degradation of methyl red was monitored by UV‐vis spectrophotometry at 430 nm. Error bars are a result of duplicates. Duplicates are from two independent activity assays with enzymes derived from the same synthesis batch.

All putative azoreductase genes were expressed with the PUREfrex2.0 system under the previously determined conditions (20 °C, 20 h) and applied in the subsequent activity assay. In addition to NADH, NADPH and the artificial representative BNAH were now used in this setup to elucidate the preferred electron donor of the enzymes (Figure 5).

Large differences in volumetric activity were detected for the azoreductases. Two of the already described enzymes, *Ecol*AzoR and *Paer*AzoR, did not show any activity with NADH or NADPH in this setup. *Paer*AzoR catalyzed the degradation of methyl red using BNAH as co‐substrate. In the case of *Ecol*AzoR, the enzyme may not have been synthesized at all or may have been synthesized too strongly, so it was present only in an insoluble form. SDS‐PAGE analysis enabled no evidence due to the small amounts formed and a superposition of the kit‐specific enzymes (Figure S3). Nevertheless, all undescribed enzymes catalyzed the degradation of methyl red and can therefore be referred to as azoreductases. NADH is the preferred electron donor in most cases, which is consistent with previous research on azoreductases.[^21^, ^31^, ^33^] Only the enzyme *Tvar*AzoR showed higher activity in combination with NADPH than with NADH. Moreover, the co‐substrate mimic BNAH was accepted by all active azoreductases and showed for *Rfer*AzoR and *Tvar*AzoR higher activities than in combination with the natural co‐substrates. Noteworthy are the very high activities of *Styp*AzoR and *Efae*AzoR with 851±102 mU mL^−1^ and 425±28 mU mL^−1^, respectively. While *Styp*AzoR showed these high activities with all electron donors, *Efae*AzoR seems to have a strong preference for NADH.

### Co‐substrate utilization and unproductive oxidation of the electron donors

**Figure 6:**
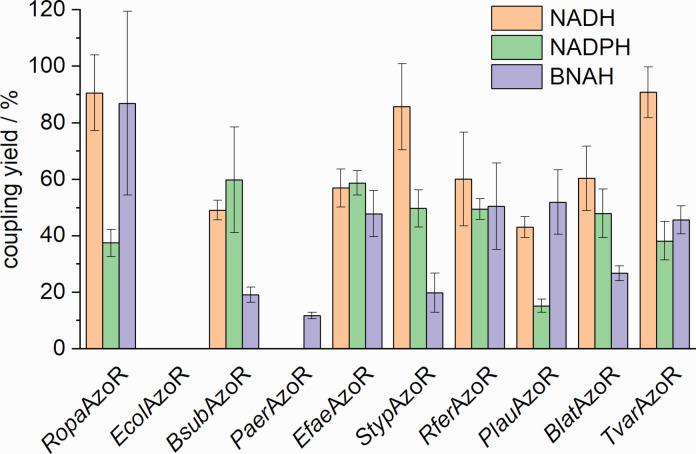
Coupling yields of electron donor and substrate consumption for the selected azoreductases. Activity assays were carried out in a 96‐well microplate with a total volume of 100 μL, consisting of 1 to 10 % (v/v) CFPS‐reaction solution and the assay solution with following final concentrations: 100 mM sodium phosphate buffer (pH 6), 50 μM FMN, 30 μM methyl red, and 150 μM NADH/NADPH/BNAH. Reactions were incubated at 25 °C and the degradation of methyl red was monitored by UV‐vis spectrophotometry at 430 nm. Consumption of the co‐substrates were also followed at their respective wavelengths (NADH 340 nm, NADPH 340 nm, and BNAH 358 nm). Error bars are a result of duplicates. Duplicates are from two independent activity assays with enzymes derived from the same synthesis batch.

To determine the efficiency of co‐substrate utilization, the coupling yields were calculated (Equation S1), defined as the yield of electrons used for substrate reduction that originate from the electron donor (Figure 6), as a previous study reported decoupling and unproductive NADH oxidation for azoreductases.^[9]^

Nonspecific co‐substrate oxidation was also detected in the case of all the enzymes investigated. The highest coupling yield was achieved with *Tvar*AzoR in the case of NADH with about 91 %. In general, NADH appears to be not only the preferred electron donor for most azoreductases but is also used most efficiently. However, for some azoreductases the coupling yield of BNAH is comparable with the coupling yields for the natural co‐substrates. This makes these enzymes particularly interesting for further applications, since they do not only accept a less expensive synthetic co‐substrate analogue, but also use it with similar efficiency, so that only comparatively small amounts are required for the reaction.

### Expanding the screening for further substrates

**Figure 7:**
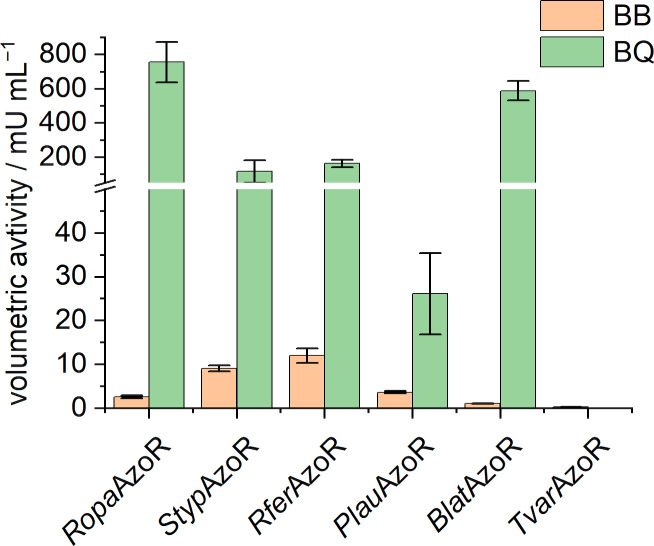
Volumetric activities for the selected azoreductases for Brilliant Black (BB) and *p*‐benzoquinone (BQ). Activity assays were carried out in a 96‐well microplate with a total volume of 100 μL, consisting of 1 to 10 % (v/v) CFPS‐reaction solution and the assay solution with following final concentrations: 100 mM sodium phosphate buffer (pH 6), 50 μM FMN, 30 μM Brilliant Black/60 μM *p*‐benzoquinone, and 150 μM NADH. Reactions were incubated at 25 °C and the degradation of BB was monitored by UV‐vis spectrophotometry at 570 nm. The activity of the BQ reduction is calculated via the NADH consumption, which was monitored at 340 nm. Error bars are a result of duplicates. Duplicates are from two independent activity assays with enzymes derived from the same synthesis batch.

The here described screening setup can easily be extended to other dyes or other possible substrates. The selected yet undescribed enzymes are annotated as potential (FMN)‐dependent NADH : quinone oxidoreductases. Therefore, we decided to also check the acceptability of a quinone, namely *p*‐benzoquinone (BQ). Furthermore, the more complex and bulkier azo dye Brilliant Black (BB), a synthetic diazo dye used in food industries, was selected as a further substrate. *Ropa*AzoR was selected additionally as a positive control, as it is already described as a BQ reducing and BB degrading enzyme.[^12^, ^21^] The volumetric activities were determined in the aforementioned setup (Figure 7).

All five enzymes and *Ropa*AzoR were able to degrade BB. *Styp*AzoR and *Rfer*AzoR showed the highest activities of 9.1±0.7 and 12.0±1.7 mU mL^−1^, respectively. *Tvar*AzoR showed the lowest activity, however, degradation was also detected here. Compared to the values with MR as the substrate, these activities are 1 to 2 orders of magnitude lower. Furthermore, it was shown that the activity of the enzymes in combination with BQ as substrate was significantly higher and in a comparable range as with MR as substrate. For *Tvar*AzoR, no activity could be observed. Thus, it was possible to prove that four of the five enzymes are (FMN)‐dependent NADH : quinone oxidoreductases. These results also indicate the broad substrate spectrum, often including various azo dyes and quinones, already described for other azoreductases.^[4]^

The newly described enzymes partly showed high activities for different substrates with different co‐substrates and could therefore be interesting candidates for further investigations. Due to the low expression levels in CFPS, an *in vivo* synthesis of the candidates could be carried out to provide sufficient quantities of enzyme to test more specifically process‐related reaction parameters such as the conversion of high substrate concentrations or the evaluation of product titers and space‐time‐yields.

In our opinion, the developed screening setup can be applied to other putative azoreductases and can be easily adapted for a particular scientific question. Evaluation of substrate scopes, stability tests, and screening for the best reaction parameters can be performed for a large number of homologs or enzyme variants in a short time. For example, the kinetic and thermodynamic stability of *Pp*AzoR *Pseudomonas putida* MET94 was improved by directed evolution and testing of about 10,000 clones with recombination by DNA Shuffling and a mutant library construction.^[34]^ In the case of our presented screening approach, the construction of the mutant library could be neglected and the use of plasmids or even the direct use of the linear PCR products would be possible.[^35^, ^36^] Especially in comparison with cell‐based heterologous expression and purification, CFPS can offer labor and time savings. Although the synthesis time of 20 h in this study is quite long and similar to an *in vivo* synthesis, laborious steps can be avoided. Once a CFPS system is ready and a DNA template prepared, a synthesis can be performed immediately with an assay directly following. In contrast, a heterologous *in vivo* expression needs a few additional work steps and operations. First, an expression strain must be constructed, which takes at least two days, but can be used permanently afterward. For the expression, a pre‐culture must be prepared, the main culture inoculated, and the growth process monitored by sampling. In most cases, induction of expression is required. After the cultivation, a cell harvest (most likely centrifugation) must be performed and a cell disruption step is required, followed by another centrifugation step. In the case of azoreductases, purification and isolation of the enzymes must also be carried out due to the endogenous azoreductase of *E. coli*. If you neglect the strain construction, this results in an approximate duration of 34 hours and significantly more work steps than when using the CFPS. However, it should be noted that the costs of a PURE CFPS system are quite high. Therefore, it would make sense to use an *E. coli* strain for the preparation of the CFPS extract that contains a knock‐out of the endogenous azoreductase. Thus, it would be possible to carry out such a screening with a comparatively inexpensive in‐house extract‐based CFPS system.

## Conclusion

The study shows that a screening setup using CFPS in combination with a subsequent *in vitro* activity assay can be implemented for azoreductases. Five, formerly never characterized putative enzymes, were proven to be azoreductases and four of them also showed the ability to reduce quinones. Furthermore, the co‐substrate preference of all tested enzymes could be determined, showing a clear tendency toward the natural substrate NADH for the most enzymes. The artificial co‐substrate mimic BNAH was also accepted for all active azoreductases, allowing the elimination of more expensive co‐substrates in potential applications. Further research can be done by varying the substrates, expanding the set of co‐substrates, and testing variants of a specific azoreductase. Azoreductases have great potential for wastewater treatment, drug development, and biosensor assays, and the approach developed will accelerate the identification and design of potent biocatalysts for these applications.

## Experimental Section

**Plasmid constructs**: The construction of the plasmid pET16bP_AzoRo, pET16bP_AzoRo+FDH and pET16bP_FDH+AzoRo is described elsewhere.^[9]^ Codon optimized and GC content adjusted gene sequences coding for the remaining AzoR enzymes were purchased as DNA strings from Thermo Fisher Scientific (Waltham, MA, USA) and cloned into a pET‐24a(+) vector via Gibson cloning.^[37]^ All vector constructs were checked for errors with sanger sequencing (Microsynth Seqlab, Göttingen, Germany). The gene sequences, their corresponding amino acid sequences and the primer sequences can be found in Supporting information (Table S1).

**Cell‐free protein synthesis**: CFPS were performed using an in‐house *E. coli* extract‐based system and the PUREfrex2.0 system (GeneFrontier). The *E. coli* extract was prepared as described by Rolf et al.^[38]^ Extract‐based CFPS reactions with a reaction volume of 10 μL were performed in microtubes containing: *E. coli* cell‐free extract amounting to 9.6 to 14.4 mg mL^−1^ protein, 10 mM magnesium glutamate, 130 mM potassium glutamate, 1.5 mM each of 20 amino acids (except leucine), 1.25 mM leucine, 50 mM HEPES, 1.5 mM ATP and GTP, 0.9 mM CTP and UTP, 0.2 mg mL^−1^ *E. coli* tRNA, 0.26 mM CoA, 0.33 mM NAD, 0.75 mM cAMP, 0.068 mM folinic acid, 1 mM spermidine, 30 mM 3‐PGA, and 2 % PEG‐8000. CFPS with the PUREfrex2.0 system were carried out in microtubes according to the manufacturer‘s instructions with a reaction volume of 10 to 60 μL. In chaperone‐assisted syntheses, 5 % (v/v) DnaK or GroE mix (GeneFrontier) were added, respectively. All reactions were incubated in an Eppendorf® ThermoMixer® C for 4 to 20 h at 450 rpm and 20 to 37 °C. Synthesized proteins were analyzed by sodium dodecyl sulfate polyacrylamide gel electrophoresis (SDS‐PAGE) and western blot. For the analysis of the total protein fraction, 1.5 μL of the synthesis were diluted with 3.5 μL water and mixed with a 2× SDS loading buffer (100 mM Tris‐ HCl pH 6.8, 4 % SDS, 20 % glycerol, 200 mM dithiothreitol, and 0.2 % bromophenol blue). To obtain the soluble protein fraction, samples were centrifuged at 18.000×g for 10 min and treated as previously described. All samples were subsequently incubated at 95 °C for 5 min. The PageRuler unstained protein ladder (Thermo Fischer Scientific, Waltham, MA, USA) was used as the marker. The gel was stained with 1× Lumitein Protein Gel Stain (Biotium, Inc., Fremont, CA, USA) for 30 min. Afterwards, the gel was destained in water for 10 min and resulting bands were visualized using UV light. Electrophoretic transfer was performed on a Power Blotter XL (Thermo Fischer Scientific, Waltham, MA, USA) using Power Blotter Select Transfer Stacks (Thermo Fischer Scientific, Waltham, MA, USA). The gels were electroblotted for 7 min with 1.3 Amps. The membranes were rinsed three times with deionized water, blocked with blocking buffer TBS‐T (20 mM Tris, 150 mM NaCl, 0.1 % Tween‐20, pH 7.6) with 3 % bovine serum albumin for 1 h at room temperature, and then rinsed twice with TBS‐T. The membranes were incubated for 1 h with a solution of the primary antibody 6×‐His Tag Monoclonal Antibody, diluted 1 : 2,000 in TBS‐T, and then rinsed three times with TBS‐T. The membranes treated with the antibody were further incubated for 1 h with a solution of the AP‐conjugated secondary antibody goat anti‐mouse IgG (H+L), diluted 1 : 10,000 in TBS‐T, and then rinsed three times with TBS‐T. To remove Tween‐20, the membranes were rinsed with TBS (20 mM Tris, 150 mM NaCl, pH 7.6) twice. All membranes were finally stained with a 1‐step NBT/BCIP substrate solution and incubated for 15 min.

**Screening for activity**: Activity assays were performed in 96‐well microplates with a total volume of 100 μL per well, consisting of 1 to 10 % (v/v) CFPS‐reaction solution and the assay solution with following final concentrations: 100 mM sodium phosphate buffer (pH 6), 50 μM FMN, 30 μM MR, BB, or 60 μM BQ and 150 μM NADH, NADPH, or BNAH, respectively. Reactions were incubated at 25 °C in a FLUOstar® Omega multi‐mode microplate reader (BMG LABTECH) and substrate degradation was followed at their respective wavelengths (MR 430 nm, BB 570 nm). Consumption of the co‐substrates were also followed at their respective wavelengths (NADH 340 nm, NADPH 340 nm, and BNAH 358 nm). Calibration data can be found in Supporting information (Figures S4–S6). The specific activity was defined as 1 U representing the conversion of 1 μmol MR/BB/NADH/NADPH/BNAH per min. For all experiments negative controls with CFPS mix and without a DNA template were carried out and the background activities were determined (see Supporting Information). All values stated have been adjusted for this background activities. All measurements were done in duplicates.

## Conflict of interest

The authors declare no conflict of interest.

1

## Supporting information

As a service to our authors and readers, this journal provides supporting information supplied by the authors. Such materials are peer reviewed and may be re‐organized for online delivery, but are not copy‐edited or typeset. Technical support issues arising from supporting information (other than missing files) should be addressed to the authors.

Supporting Information

Supporting Information

## Data Availability

The data that support the findings of this study are available in the supplementary material of this article.

## References

[1] K. Hunger , P. Mischke , W. Rieper , R. Raue , K. Kunde , A. Engel , in Ullmann's Encyclopedia of Industrial Chemistry, Wiley-VCH, Weinheim, 2000, pp. 6–10.

[2] R. Khan , P. Bhawana , M. H. Fulekar , Rev. Environ. Sci. Bio/Technol. 2013, 12, 75–97.

[3] B. Lellis , C. Z. Fávaro-Polonio , J. A. Pamphile , J. C. Polonio , Biotechnol. Res. Innov. 2019, 3, 275–290.

[4] S. A. Misal , K. R. Gawai , Bioresour. Bioprocess. 2018, 5, 17.

[5] W. G. Levine , S. Zbaida , in Advances in Experimental Medicine and Biology, Springer, 1991, pp. 315–321. 10.1007/978-1-4684-5877-0_39 1906222

[6] V. Chalansonnet , C. Mercier , S. Orenga , C. Gilbert , BMC Microbiol. 2017, 17, 126. 28545445 10.1186/s12866-017-1033-3 PMC5445473

[7] H. Suzuki , Appl. Microbiol. Biotechnol. 2019, 103, 3965–3978. 30941462 10.1007/s00253-019-09775-2

[8] L. Han , B. Liang , World J. Microbiol. Biotechnol. 2018, 34, 141. 30203299 10.1007/s11274-018-2530-8

[9] A. C. R. Ngo , F. P. J. Schultes , A. Maier , S. N. H. Hadewig , D. Tischler , ChemBioChem 2022, 23, e202100643. 35080802 10.1002/cbic.202100643 PMC9305538

[10] J. Qi , C. E. Paul , F. Hollmann , D. Tischler , Enzyme Microb. Technol. 2017, 100, 17–19. 28284307 10.1016/j.enzmictec.2017.02.003

[11] C. E. Paul , F. Hollmann , Appl. Microbiol. Biotechnol. 2016, 100, 4773–4778. 27094184 10.1007/s00253-016-7500-1 PMC4866995

[12] A. Christina , R. Ngo , C. Juric , J. Qi , I. Bento , D. Tischler , A. C. R. Ngo , J. Qi , C. Juric , I. Bento , D. Tischler , Arch. Biochem. Biophys. 2022, 717, 109123. 35051387 10.1016/j.abb.2022.109123

[13] E. D. Carlson , R. Gan , C. E. Hodgman , M. C. Jewett , Biotechnol. Adv. 2012, 30, 1185–1194. 22008973 10.1016/j.biotechadv.2011.09.016 PMC4038126

[14] J. Rolf , K. Rosenthal , S. Lütz , Catalysts 2019, 9, 190.

[15] D. Garenne , M. C. Haines , E. F. Romantseva , P. Freemont , E. A. Strychalski , V. Noireaux , Nat. Rev. Methods Prim. 2021, 1, 49.

[16] J. Feng , C. Yang , Z. Zhao , J. Xu , J. Li , P. Li , ACS Synth. Biol. 2021, 10, 620–631. 33719397 10.1021/acssynbio.0c00618

[17] J. Rolf , P. Nerke , A. Britner , S. Krick , S. Lütz , K. Rosenthal , Catalysts 2021, 11, 1038.

[18] J. Li , T. J. Lawton , J. S. Kostecki , A. Nisthal , J. Fang , S. L. Mayo , A. C. Rosenzweig , M. C. Jewett , Biotechnol. J. 2016, 11, 212–218. 26356243 10.1002/biot.201500030

[19] N. Nuti , P. Rottmann , A. Stucki , P. Koch , S. Panke , P. S. Dittrich , Angew. Chem. Int. Ed. 2022, 61, e202114632. 10.1002/anie.202114632 PMC9303939 34989471

[20] A. D. Halleran , R. M. Murray , ACS Synth. Biol. 2018, 7, 752–755. 29120612 10.1021/acssynbio.7b00376

[21] J. Qi , M. Schlömann , D. Tischler , J. Mol. Catal. B 2016, 130, 9–17.

[22] M. Nakanishi , C. Yatome , N. Ishida , Y. Kitade , J. Biol. Chem. 2001, 276, 46394–46399. 11583992 10.1074/jbc.M104483200

[23] Y. Shimizu , T. Kanamori , T. Ueda , Methods 2005, 36, 299–304. 16076456 10.1016/j.ymeth.2005.04.006

[24] S. Ventura , A. Villaverde , Trends Biotechnol. 2006, 24, 179–185. 16503059 10.1016/j.tibtech.2006.02.007

[25] S. Falak , M. Sajed , N. Rashid , Biologia 2022, 77, 893–905.

[26] X. Jin , W. Kightlinger , S. H. Hong , Methods Protoc. 2019, 2, 28. 10.3390/mps2020028 PMC6632115 36358105

[27] L. A. Ryabova , D. Desplancq , A. S. Spirin , A. Plückthun , Nat. Biotechnol. 1997, 15, 79–84. 9035111 10.1038/nbt0197-79

[28] X. Jiang , Y. Ookubo , I. Fujii , H. Nakano , T. Yamane , FEBS Lett. 2002, 514, 290–294. 11943168 10.1016/s0014-5793(02)02383-9

[29] S. Murakami , R. Matsumoto , T. Kanamori , Sci. Rep. 2019, 9, 671. 30679500 10.1038/s41598-018-36691-8 PMC6345822

[30] A. Bateman , M.-J. Martin , S. Orchard , M. Magrane , R. Agivetova , S. Ahmad , E. Alpi , E. H. Bowler-Barnett , R. Britto , B. Bursteinas , H. Bye-A-Jee , R. Coetzee , A. Cukura , A. Da Silva , P. Denny , T. Dogan , T. Ebenezer , J. Fan , L. G. Castro , P. Garmiri , G. Georghiou , L. Gonzales , E. Hatton-Ellis , A. Hussein , A. Ignatchenko , G. Insana , R. Ishtiaq , P. Jokinen , V. Joshi , D. Jyothi , A. Lock , R. Lopez , A. Luciani , J. Luo , Y. Lussi , A. MacDougall , F. Madeira , M. Mahmoudy , M. Menchi , A. Mishra , K. Moulang , A. Nightingale , C. S. Oliveira , S. Pundir , G. Qi , S. Raj , D. Rice , M. R. Lopez , R. Saidi , J. Sampson , T. Sawford , E. Speretta , E. Turner , N. Tyagi , P. Vasudev , V. Volynkin , K. Warner , X. Watkins , R. Zaru , H. Zellner , A. Bridge , S. Poux , N. Redaschi , L. Aimo , G. Argoud-Puy , A. Auchincloss , K. Axelsen , P. Bansal , D. Baratin , M.-C. Blatter , J. Bolleman , E. Boutet , L. Breuza , C. Casals-Casas , E. de Castro , K. C. Echioukh , E. Coudert , B. Cuche , M. Doche , D. Dornevil , A. Estreicher , M. L. Famiglietti , M. Feuermann , E. Gasteiger , S. Gehant , V. Gerritsen , A. Gos , N. Gruaz-Gumowski , U. Hinz , C. Hulo , N. Hyka-Nouspikel , F. Jungo , G. Keller , A. Kerhornou , V. Lara , P. Le Mercier , D. Lieberherr , T. Lombardot , X. Martin , P. Masson , A. Morgat , T. B. Neto , S. Paesano , I. Pedruzzi , S. Pilbout , L. Pourcel , M. Pozzato , M. Pruess , C. Rivoire , C. Sigrist , K. Sonesson , A. Stutz , S. Sundaram , M. Tognolli , L. Verbregue , C. H. Wu , C. N. Arighi , L. Arminski , C. Chen , Y. Chen , J. S. Garavelli , H. Huang , K. Laiho , P. McGarvey , D. A. Natale , K. Ross , C. R. Vinayaka , Q. Wang , Y. Wang , L.-S. Yeh , J. Zhang , P. Ruch , D. Teodoro , Nucleic Acids Res. 2021, 49, D480–D489. 33237286 10.1093/nar/gkaa1100 PMC7778908

[31] Y. Nishiya , Y. Yamamoto , Biosci. Biotechnol. Biochem. 2007, 71, 611–614. 17284825 10.1271/bbb.60548

[32] C.-J. Wang , C. Hagemeier , N. Rahman , E. Lowe , M. Noble , M. Coughtrie , E. Sim , I. Westwood , J. Mol. Biol. 2007, 373, 1213–1228. 17904577 10.1016/j.jmb.2007.08.048

[33] H. Chen , R.-F. Wang , C. E. Cerniglia , Protein Expression Purif. 2004, 34, 302–310. 10.1016/j.pep.2003.12.016 PMC5875116 15003265

[34] V. Brissos , N. Gonçalves , E. P. Melo , L. O. Martins , PLoS One 2014, 9, e87209. 24475252 10.1371/journal.pone.0087209 PMC3903626

[35] L. T. Quertinmont , R. Orru , S. Lutz , Chem. Commun. 2015, 51, 122–124. 10.1039/c4cc08240k 25384037

[36] M. A. McSweeney , M. P. Styczynski , Front. Bioeng. Biotechnol. 2021, 9, 715328. 34354989 10.3389/fbioe.2021.715328 PMC8329657

[37] D. G. Gibson , L. Young , R.-Y. Chuang , J. C. Venter , C. A. Hutchison , H. O. Smith , Nat. Methods 2009, 6, 343–345. 19363495 10.1038/nmeth.1318

[38] J. Rolf , R. Siedentop , S. Lütz , K. Rosenthal , Int. J. Mol. Sci. 2019, 21, 105. 10.3390/ijms21010105 PMC6981698 31877895

