# Supplementary material for: Cell‐Free Protein Synthesis for the Screening of Novel Azoreductases and Their Preferred Electron Donor
Source: Chembiochem. 2022 Jun 16;23(15):e202200121. doi: 10.1002/cbic.202200121 (PMC9401864; doi:10.1002/cbic.202200121)
Supplement: Supplementary file 1 — Supporting Information [file CBIC-23-0-s002.pdf]

ChemBioChem

Supporting Information

Cell-Free Protein Synthesis for the Screening of Novel Azoreductases and Their Preferred Electron Donor

Jascha Rolf, Anna Christina Reyes Ngo, Stephan Lütz, Dirk Tischler, and Katrin Rosenthal*

Table of contents

Figure S1: Multiple sequence alignment of azoreductases from different species.

Figure S2: Maximum Likelihood distance tree of azoreductases.

Figure S3: SDS-PAGE gel image of cell-free synthesized azoreductases at 20 °C for 20 h with PUREfrex2.0.

Figure S4: Standard curve of methyl red.

Figure S5: Standard curve of brilliant black.

Figure S6: Standard curves of co-substrates.

Equation S1: Coupling yield of electron donor and substrate consumption.

Data, DNA sequences, amino acid sequences, and primer sequences are shared in a separate excel file.

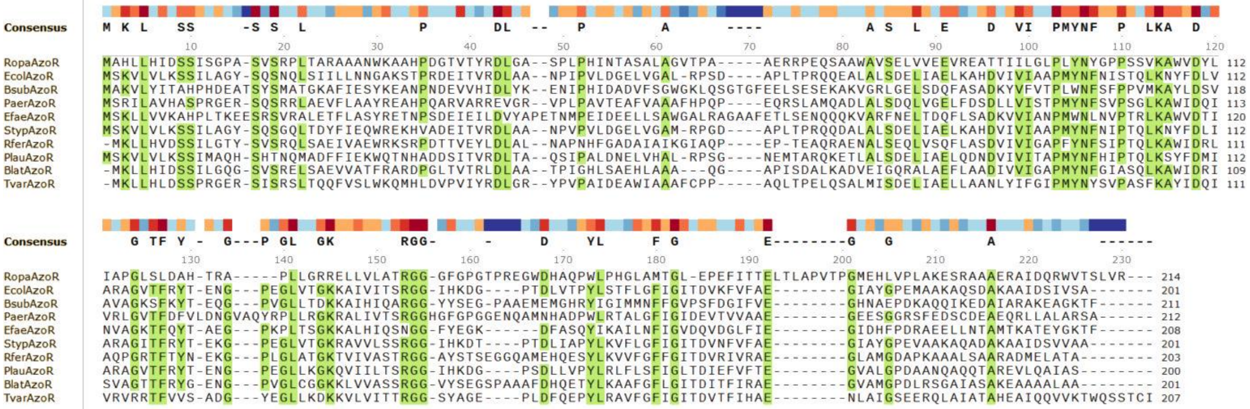

Figure S1: Multiple sequence alignment of azoreductases from different species. Shading indicates a consensus sequence above a threshold of 75%. The alignment was generated using MUSCLE (SnapGene®®, GSL Biotech LLC, Chicago, IL, USA, Version 4.3.11) [1].

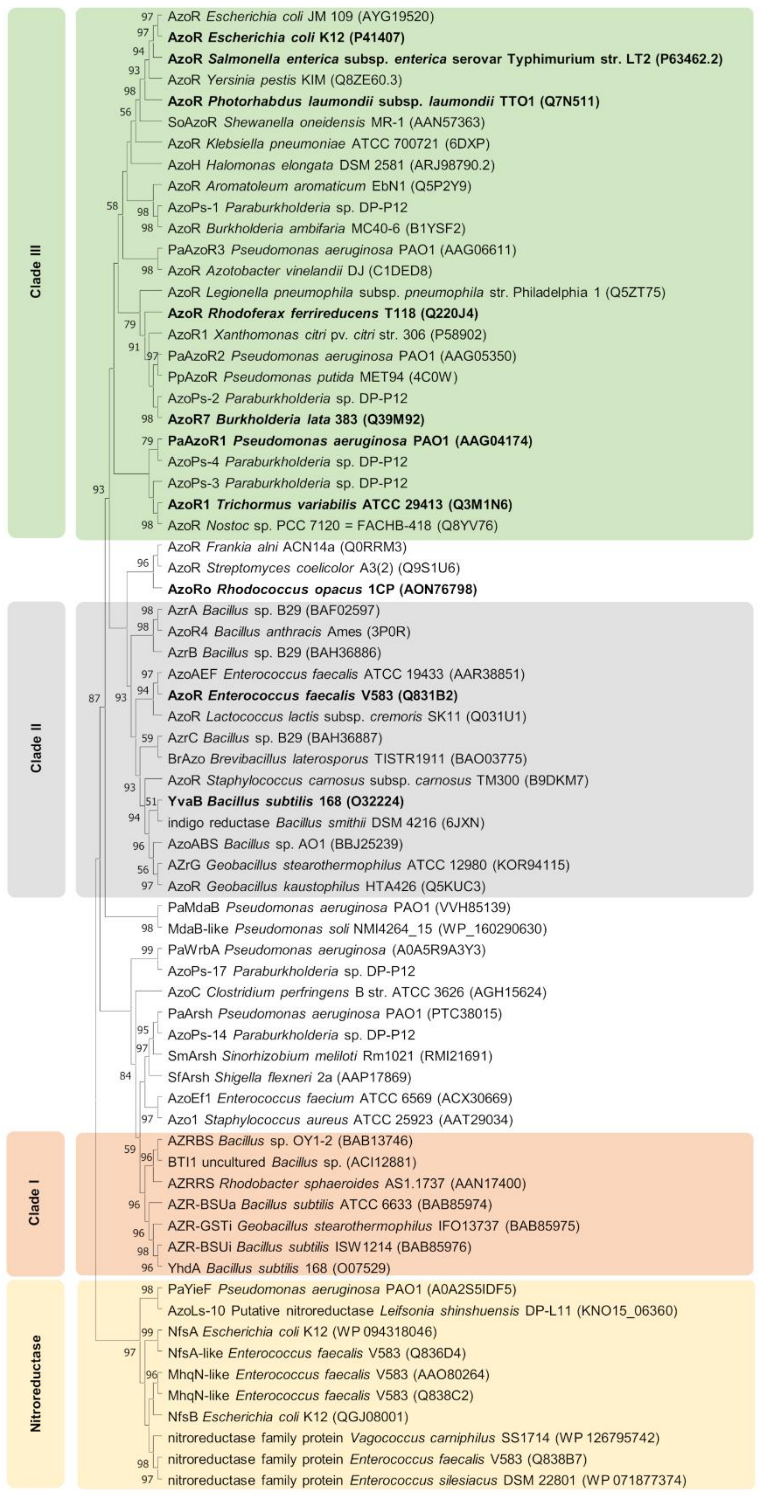

Figure S2: Maximum Likelihood distance tree of azoreductases. The phylogenetic tree was constructed on base of the earlier presented Minimum-Evolution tree employing amino acid sequences (here 70) [2]. The Maximum Likelihood method and JTT matrix-based model were employed to generate the tree by means of MEGA11 [3,4]. The tree with the highest log likelihood (-22200.09) is shown. Numbers at nodes indicate the bootstrap-value from 1000 replicates as percentage. The bootstrap values below 50% are not shown. Sequences studied herein are marked as bold. Nitroreductases have been used to root the distance tree. The 3 clades of flavin-dependent azoreductases are highlighted.

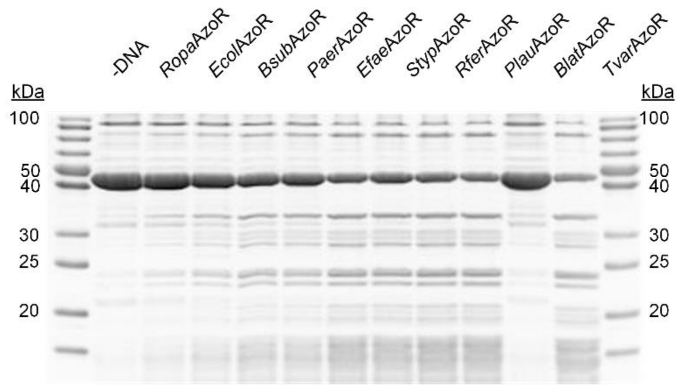

Figure S3: SDS-PAGE gel image of cell-free synthesized azoreductases at 20 °C for 20 h with PUREfrex2.0. -DNA: CFPS mix without plasmid template. *Ropa*AzoR: 25.3 kDa (with His-tag); *Ecol*AzoR: 21.4 kDa; *Bsub*AzoR: 23.3 kDa; *Paer*AzoR: 23.1 kDa; *Efae*AzoR: 23.2 kDa; *Styp*AzoR: 21.6 kDa; *Rfer*AzoR: 21.7 kDa; *Plau*AzoR: 22.1 kDa; *Blat*AzoR: 20.7 kDa; *Tvar*AzoR: 23.1 kDa.

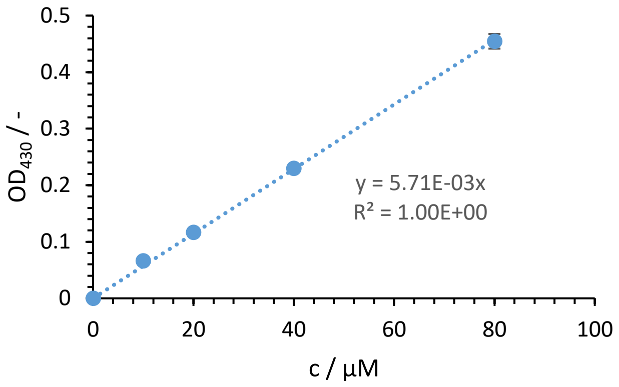

Figure S4: Standard curve of methyl red for spectrophotometry measurements in a FLUOstar®® Omega multi-mode microplate reader (BMG LABTECH) at 430 nm.

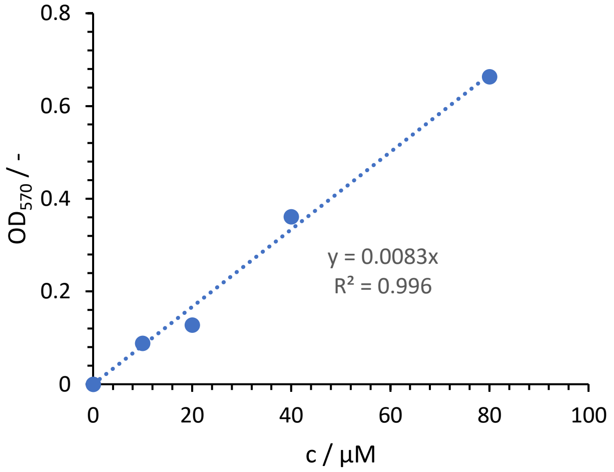

Figure S5: Standard curve of brilliant black for spectrophotometry measurements in a FLUOstar®® Omega multi-mode microplate reader (BMG LABTECH) at 570 nm.

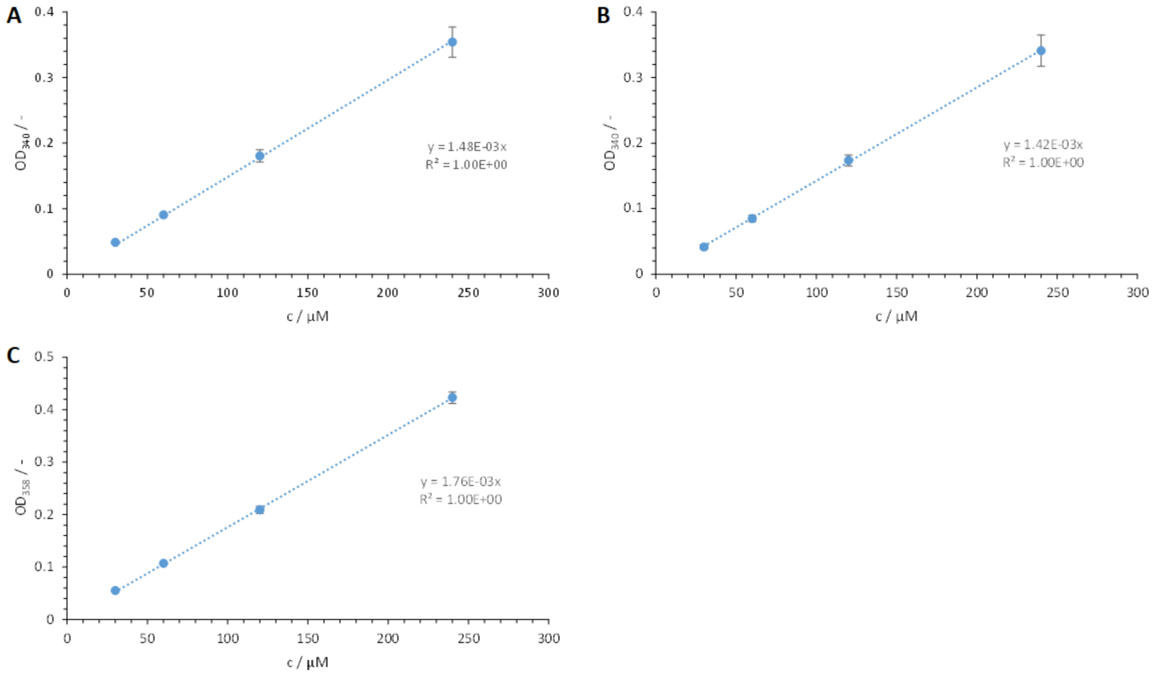

Figure S6: Standard curves of co-substrates for spectrophotometry measurements in a FLUOstar®® Omega multi-mode microplate reader (BMG LABTECH) at their respective wavelengths: **A** NADH 340 nm, **B** NADPH 340 nm, **C** BNAH 358 nm

Equation S1:

$$CY = \frac{a_{MR}}{2 \cdot a_{co\text{-}substrate}} \cdot 100\%$$

CY: coupling yield [%]

a_i: activity based on substrate i [U]

Coupling yield is defined as the yield of electrons employed for substrate reduction which were derived of the co-substrate. (Factor 2 occurs because two molecules of co-substrate are needed per molecule of substrate for the reduction)

References

1. Edgar, R.C. MUSCLE: Multiple Sequence Alignment with High Accuracy and High Throughput. *Nucleic Acids Res.* **2004**, *32*, 1792–1797, doi:10.1093/nar/gkh340.
2. Kumaran, S.; Ngo, A.C.R.; Schultes, F.P.J.; Saravanan, V.S.; Tischler, D. In Vitro and in Silico Analysis of Brilliant Black Degradation by Actinobacteria and a Paraburkholderia Sp. *Genomics* **2022**, *114*, 110266, doi:10.1016/j.ygeno.2022.01.003.
3. Jones, D.T.; Taylor, W.R.; Thornton, J.M. The Rapid Generation of Mutation Data Matrices from Protein Sequences. *Bioinformatics* **1992**, *8*, 275–282, doi:10.1093/bioinformatics/8.3.275.
4. Tamura, K.; Stecher, G.; Kumar, S. MEGA11: Molecular Evolutionary Genetics Analysis Version 11. *Mol. Biol. Evol.* **2021**, *38*, 3022–3027, doi:10.1093/molbev/msab120.
